# Tools for classification of growing/non-growing bacterial colonies using laser speckle imaging

**DOI:** 10.3389/fmicb.2023.1279667

**Published:** 2023-10-20

**Authors:** Ilya Balmages, Janis Liepins, Stivens Zolins, Dmitrijs Bliznuks, Renars Broks, Ilze Lihacova, Alexey Lihachev

**Affiliations:** ^1^Faculty of Computer Science and Information Technology, Riga Technical University, Riga, Latvia; ^2^Institute of Atomic Physics and Spectroscopy, University of Latvia, Riga, Latvia; ^3^Institute of Microbiology and Biotechnology, University of Latvia, Riga, Latvia; ^4^Laboratorija Auctoritas Ltd., Riga, Latvia; ^5^Department of Biology and Microbiology, Riga Stradins University, Riga, Latvia

**Keywords:** laser speckle imaging, sensitive subpixel correlation method, microorganism activity estimation, image processing, artificial neural network

## Abstract

Prior research has indicated the feasibility of assessing growth—associated activity in bacterial colonies through the application of laser speckle imaging techniques. A subpixel correlation method was employed to identify variations in sequential laser speckle images, thereby facilitating the visualization of specific zones indicative of microbial growth within the colony. Such differentiation between active (growing) and inactive (non-growing) bacterial colonies holds considerable implications for medical applications, like bacterial response to certain drugs or antibiotics. The present study substantiates the capability of laser speckle imaging to categorize bacterial colonies as growing or non-growing, a parameter which nonvisible in colonies when observed under white light illumination.

## 1. Introduction

Assessing bacterial growth constitutes a cornerstone in the study of bacterial physiology and its regulatory mechanisms. Such evaluations serve dual purposes: they augment the understanding of microbiological processes and facilitate the prediction of growth rates pivotal for biotechnological applications (Atolia et al., 2020). Numerous industries rely on bacterial metabolism and by—products for the synthesis of pharmaceuticals, food, and various industrial commodities. The rate of bacterial growth and by—product formation is subject to modulation by nutritional and environmental parameters (Kouzuma and Watanabe, 2014). Hence, there is a pressing need for rapid and efficient methods to optimize existing procedures and to innovate new techniques.

Like all matter, bacterial colonies and their growth environments possess distinct optical properties, including scattering and absorption. Laser illumination of such surfaces reveals that alterations in these optical properties yield temporal variations in the ensuing speckle patterns (Goodman, 2007). Changes in speckle patterns can be attributable to intrinsic shifts in the object’s properties or to external perturbations—such as the proliferation of bacterial colonies on the surface under observation. Consequently, laser speckle imaging can detect the activity and growth of bacterial colonies.

The utility of laser speckle imaging extends beyond mere colony detection (Balmages et al., 2020). Subsequent studies have revealed the capacity of this technique to register colony activity (Balmages et al., 2021b) and correlate it with bacterial proliferation (Balmages et al., 2021a, 2023b). In biological tests centered on microbial colony growth analysis, the ability to detect when a colony ceases to grow can be important.

Laser speckle imaging can detect and monitor microbial colonies, but without additional image processing, it lacks the capability to visualize zones of activity (growth). It also falls short in real-time detection of changes in microbial growth within the colony, including pinpointing the moment the colony becomes inactive. Although the colony is observed, its activity status: growing or non-growing remains unknown. To distinguish areas with and without colonies, a change in the statistical properties of the environment suffices. To further categorize an observed bacterial colony as either growing or non-growing, the study employs speckle subpixel correlation analyses. The objective of this analysis is to detect subtle alterations indicative of microbial growth within the colony.

In our study, we use two types of data (1) laser speckle imaging without the utilization of a subpixel correlation method, serving to identify the presence of a colony, and (2) laser speckle imaging integrated with a sensitive subpixel correlation method, which ascertains the colony’s activity status, whether it is growing or not. Utilizing these datasets, both the location and the growth status of the microbial colony can be determined. Additionally, the subpixel correlation analysis facilitates the identification of the moment at which the colony ceases to grow. This analytical capability is instrumental for investigating the influence of chemical agents, such as drugs and antibiotics, on the dynamics of colony growth.

For the purpose of automating the classification of colonies as either “growing” or “non-growing,” various algorithmic tools may be employed. These may include Linear Classifiers, Logistic Regression, Quadratic Classifiers, and Bayes Classifiers, among others. In this investigation, artificial neural networks (ANNs) were utilized, leveraging their inherent advantages of massively parallel structure and generalization capabilities. ANNs also offer the added benefit of rejecting ambiguous patterns, thereby enhancing classification performance (Haykin, 2009).

## 2. Materials and methods

### 2.1. The experimental setup

**Figure 1:**
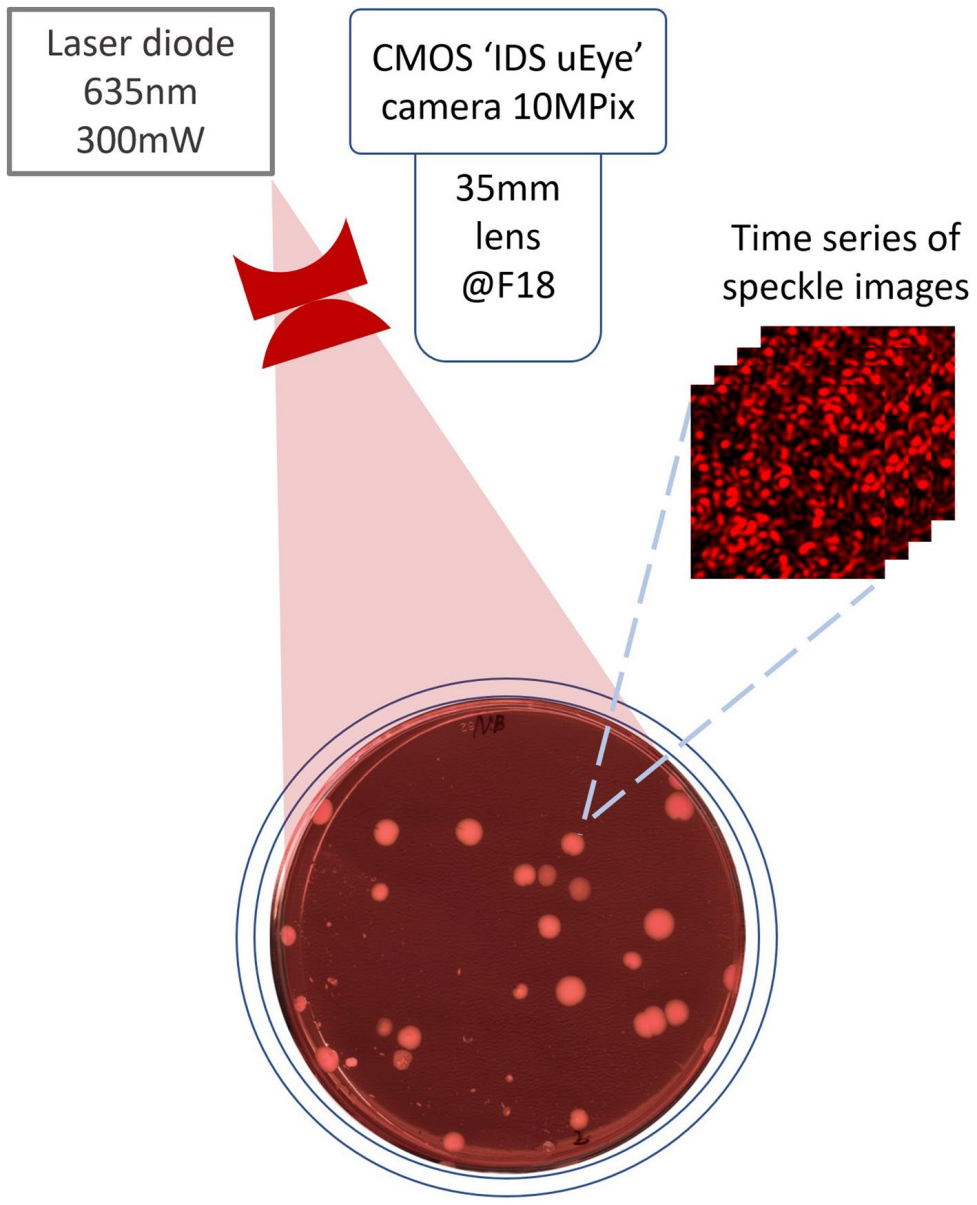
Setup scheme for speckle image capturing.

For the purposes of this research, the experimental apparatus consisted of a laser speckle imaging system equipped with a linearly polarized 635 nm multimode diode—pumped solid—state laser, capable of outputting power up to 300 mW and having a coherence length of 30 cm. The optical assembly also included a 35 mm C—mount lens set at F18 with a magnification factor of 0.2, an optical attenuator, and an IDS CMOS 10 MPix camera (Balmages et al., 2023a). The optical attenuator served to optimize exposure for image capture while mitigating heating effects on the illuminated agar plate. The power density of the scattered laser light on the agar plate’s surface was constrained to not exceed 3–5 mW/cm^2^. Image capture proceeded at 30 s intervals over a duration of 107 h, with an exposure time set to 1 s for each frame. The layout of the experimental setup is available on the Figure 1.

### 2.2. Microbial strains and cultivation conditions

In this study, bacteria *Vibrio natriegens* DSM 759 from the German Collection of Microorganisms and Cell Cultures GmbH were used. *Vibrio natriegens* were cultivated at 30°C temperature on NB salt agar (grams per liter: Difco Nutrient Broth 8 g, NaCl 15 g), as suggested by Weinstock et al. (2016). To observe colony growth, preculture in NB liquid media was inoculated from a single colony and cultivated for 12 h; preculture was diluted to approximately 200–400 cells per mL, then 50 μL were evenly streaked out on NB agar in standard Petri dish.

### 2.3. Conversion of laser speckle image into time signals: description of the algorithm

In the experimental design, the field of view was partitioned into smaller segments, each comprising N × N pixels. Subsequent analytical procedures were executed independently for each of these segments. A two-dimensional normalized cross-correlation was conducted between sequential N × N image fragments over the duration of the experiment, as described by Gåsvik (2002) and represented by Equation (1).


(1) $$\begin{array}{l} NCC\left(u,v\right)=\\ \;\frac{\sum_{x}\sum_{y}\left(\left(a\left(x,y\right)-\bar{a}\right)\cdot \left(b\left(x-u,y-v\right)-\bar{b}\right)\right)}{\sqrt{\sum_{x}\sum_{y}{\left(a\left(x,y\right)-\bar{a}\right)}^{2}\cdot \sum_{x}\sum_{y}{\left(b\left(x-u,y-v\right)-\bar{b}\right)}^{2}}} \end{array}$$

Where *a*(*x*,*y*) and *b*(*x*,*y*) are two adjacent frames in a sequence, $\bar{a}$ and $\bar{b}$ are the average values of these two frames, *u* and *v* are spatial displacement between frames *a*(*x*,*y*) and *b*(*x*,*y*) in the directions of *x* and *y*, respectively.

The correlation peak shift characterizes the changes that occur between consecutive N × N pixels images [Equation (2)].


(2) $$\left(\hat{u},\hat{v}\right)=\underset{u,v}{\arg\max}\left(NCC\left(u,v\right)\right)$$

Parabolic interpolation around the correlation peak makes it possible to find a more accurate offset [Lai and Torp, 1999; Equation (3)].


(3) $$\begin{array}{l} {\hat{\delta }}_{x}=-\frac{b_{u}}{2a_{u}}=\\ \;\frac{NCC\left(\hat{u}-1,\hat{v}\right)-NCC\left(\hat{u}+1,\hat{v}\right)}{2\left(NCC\left(\hat{u}-1,\hat{v}\right)-2NCC\left(\hat{u},\hat{v}\right)+NCC\left(\hat{u}+1,\hat{v}\right)\right)} \end{array}$$

where $a_{u}$ and $b_{u}$ are the coefficients of the parabola.

The offsets obtained between each pair of adjacent images were accumulated [Equation (4)].


(4) $$sig\left[n\right]=\sum_{i=1}^{n}\hat{\delta }\left[i\right]$$

Thus, each section of the N × N pixels image was converted into a “time signal.”

This algorithm was performed for all N × N pixel sections of the experiment field. Thus, obtained an array from the “time signals.”

To find local extrema and to avoid the influence of local transient spikes, it is worth smoothing the signal (Tukey, 1974). The signal envelope function was used. A moving root-mean-square technique or other similar algorithm can be used for this purpose:


(5) $$Env\left[n\right]=\sqrt{\frac{1}{N}\sum_{k=n-N+1}^{n}sig{\left[k\right]}^{2}}$$

Where *N* is the length of the window, *n* is the current sample, *k* is the index running inside the window. Accordingly, *N*—the length of the window is responsible for how much stronger the signal will be smoothed. To avoid outliers when performing the RMS technique, the extreme values can be truncated, as it is done in the truncated mean technique (Lehmann, 1983).

## 3. Results

### 3.1. Colony analysis with laser speckle technique without additional processing

Marker-free imaging is typically used for registration of microbial colony growth under white light illumination. Such methods furnish information about the location of the Colony Forming Unit (CFU) as well as its rate of growth. However, these approaches are not particularly elucidative concerning growth within colony. As indicated by Pirt (1967), colony growth is primarily driven by microbial proliferation at the colony’s periphery. The role of subpixel correlation analysis, as outlined by Balmages et al. (2022), is to document such intracolonial microbial growth activity.

Furthermore, images procured via laser speckle techniques, when unaccompanied by subpixel correlation analysis, are insufficient for observing the ramifications of internal changes within the colonies (Balmages et al., 2021a, 2023b).

**Figure 2:**
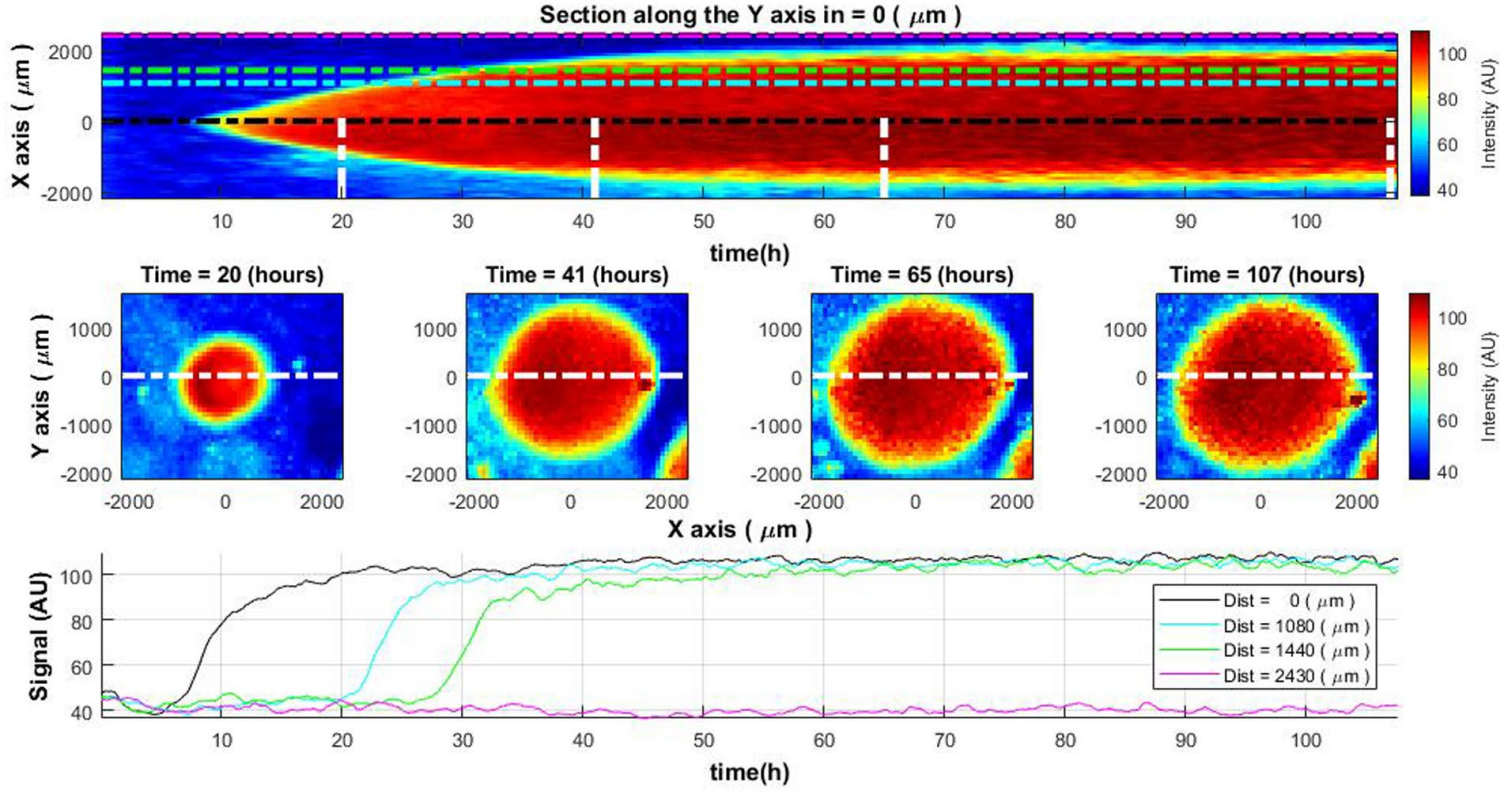
Spatiotemporal distribution of the laser speckle reflectance intensity across *Vibrio natriegens* colony. The change of colony cross-sectional intensity over time (top) and colony size at specific time moments (middle row). The white lines in the top image define the time of the cross-section demonstration in the middle row images. Lines of different colors in the top and bottom images indicate the measurement of intensity level at the center of the colony and at different distances from the colony center (bottom).

To demonstrate the results of laser speckle imaging without additional processing, one may consider a case study involving the measurement of a bacterial colony recorded over a span exceeding 100 h. In the initial 45–50 h, the colony exhibits active growth. Subsequent to this phase, growth arrest occurs, presumably owing to nutrient depletion as a consequence of the expansion of the colony and the proximity of adjacent colonies. Examination of the colony’s cross-sectional diameter as a function of time reveals certain growth dynamics (refer to Figure 2, top and middle rows). Initially, bacterial cells are observed at the colony’s center, following which they manifest at increasing distances from the center. Upon attaining a specific diameter, the colony ceases to grow. While the images confirm the bacterial presence throughout the occupied diameter, they fail to elucidate the specific physiological state of the bacteria, i.e., whether they are in a state of growth or stasis.

Further insights can be gained by analyzing the curves representing intensity levels at varying distances from the colony’s center (Figure 2, bottom row). These curves depict an increment in intensity at distinct temporal intervals, contingent on the distance from the center. Once a specific intensity level is reached, it remains relatively constant. This information, however, does not furnish sufficient evidence to deduce the ongoing biological activities within the colony.

### 3.2. Laser speckle technique with subpixel correlation analysis reveals growth changes within the colony

Alterations in the growth dynamics within a bacterial colony—whether spatial movement, increasing activity, or halt of the proliferation—can be effectively discerned through the integration of laser speckle imaging techniques with subpixel correlation analysis. Consequently, if a bacterial colony cease to grow at a certain juncture, this cessation is readily detectable. Such a halt in growth could be attributable to factors such as nutrient depletion in the media (Cooper et al., 1968; Chacón et al., 2018) or the impact of external variables, like presence of inhibiting compounds, including antibiotics.

**Figure 3:**
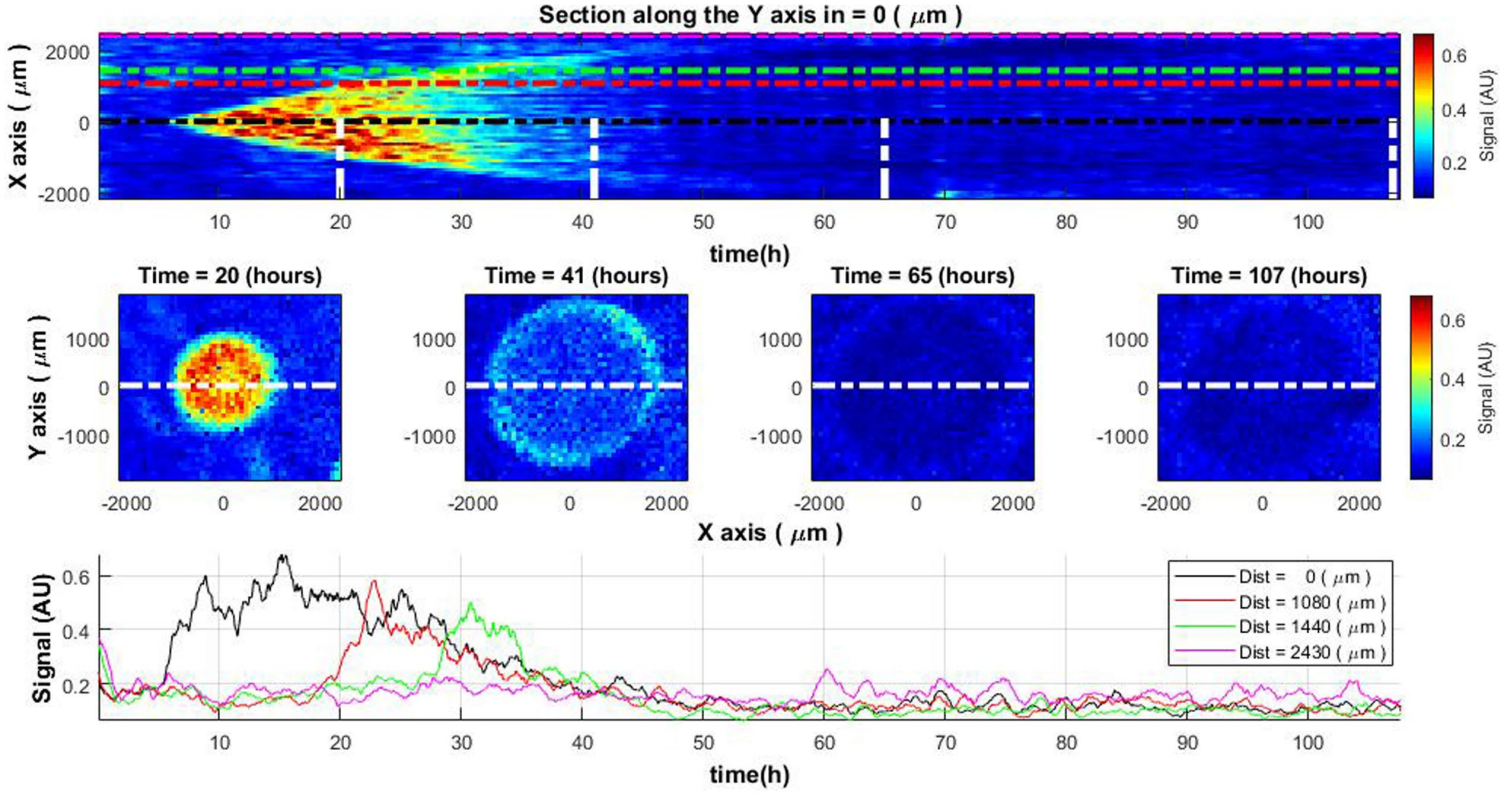
Spatiotemporal distribution of the activity signal across *Vibrio natriegens* colony. The change of colony cross-sectional activity signal over time (top) and colony activity signal at specific time moments (middle row). The white lines in the top image define the time of the cross-section demonstration in central images. Lines of different colors in the top and bottom images indicate the measurement signals at the center of the colony and at different distances from the colony center (bottom).

Upon conducting subpixel correlation analysis, one can observe specific growth patterns within the colony across its diameter as a function of time (refer to Figure 3, top and middle rows). Initially, bacterial activity is localized at the colony’s center, expanding radially over time. Observations indicate that once a specific diameter is attained, growth arrests. Concurrently, the associated signal indicative of bacterial growth begins to attenuate and ultimately vanishes. Understanding that the signal is indicative of bacterial growth, it becomes clear that the growth of the colony has ceased.

Examining signal curves along the temporal axis at various distances from the center of the colony (Figure 3, bottom), one notes that bacterial growth (manifested as high signal values) migrates outward from the center. At first, the strongest signal is observed at the center and diminishes as the distance from the center increases. Furthermore, the signal diminishes over time and vanishes approximately 45–50 h into the initiation of the experiment, corresponding to the cessation of bacterial growth within the colony.

**Figure 4:**
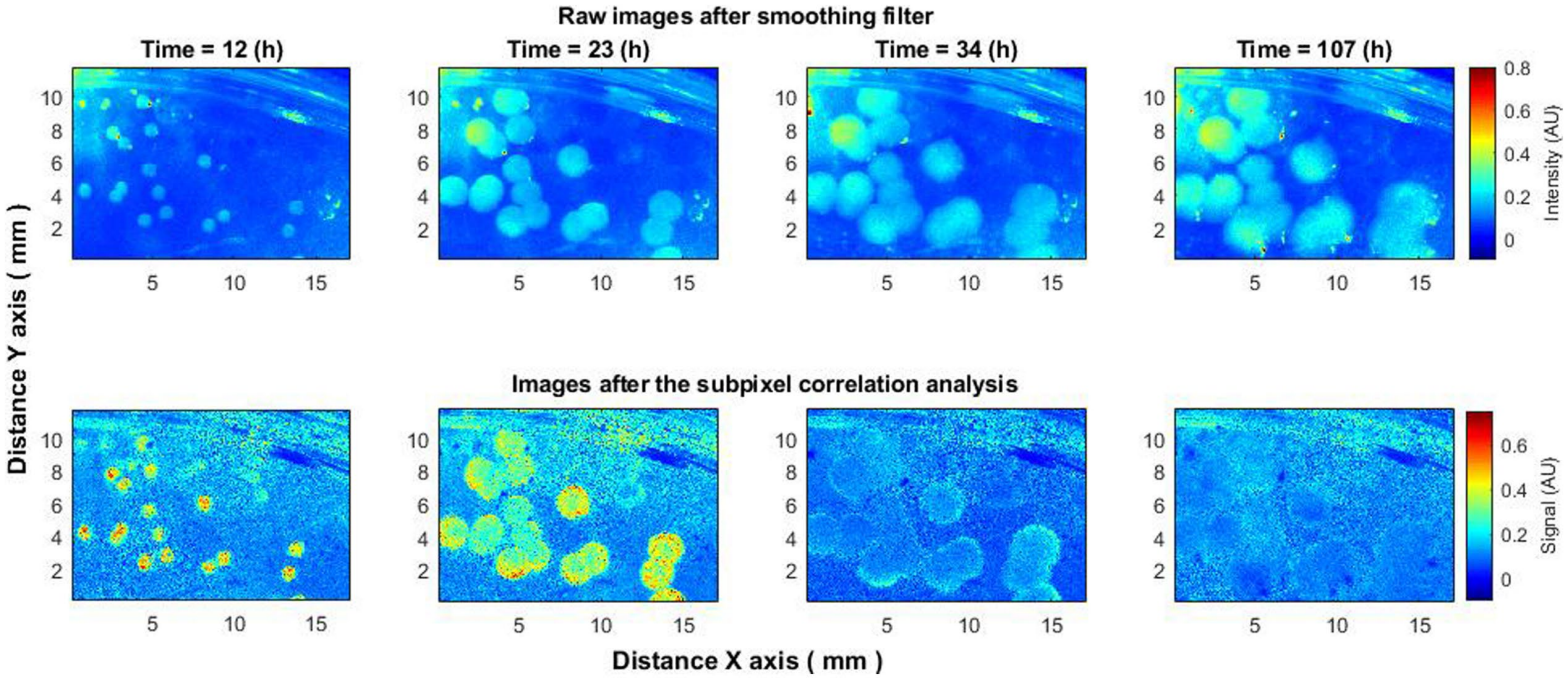
Laser speckle reflectance intensity images (top) and corresponding activity signal images (bottom) captured from 15 to 20 *Vibrio natriegens* colonies at various time points during the incubation period.

To verify the consistency of the aforementioned effect, analyses were conducted on 15–20 bacterial colonies. Analogous behavior was discerned across all colonies. Elevated activity was observed in the initial hours of the experiment (Figure 4), followed by the “ring” effect reported previously by Balmages et al. (2021a, 2023b), after which activity ceased.

**Figure 5:**
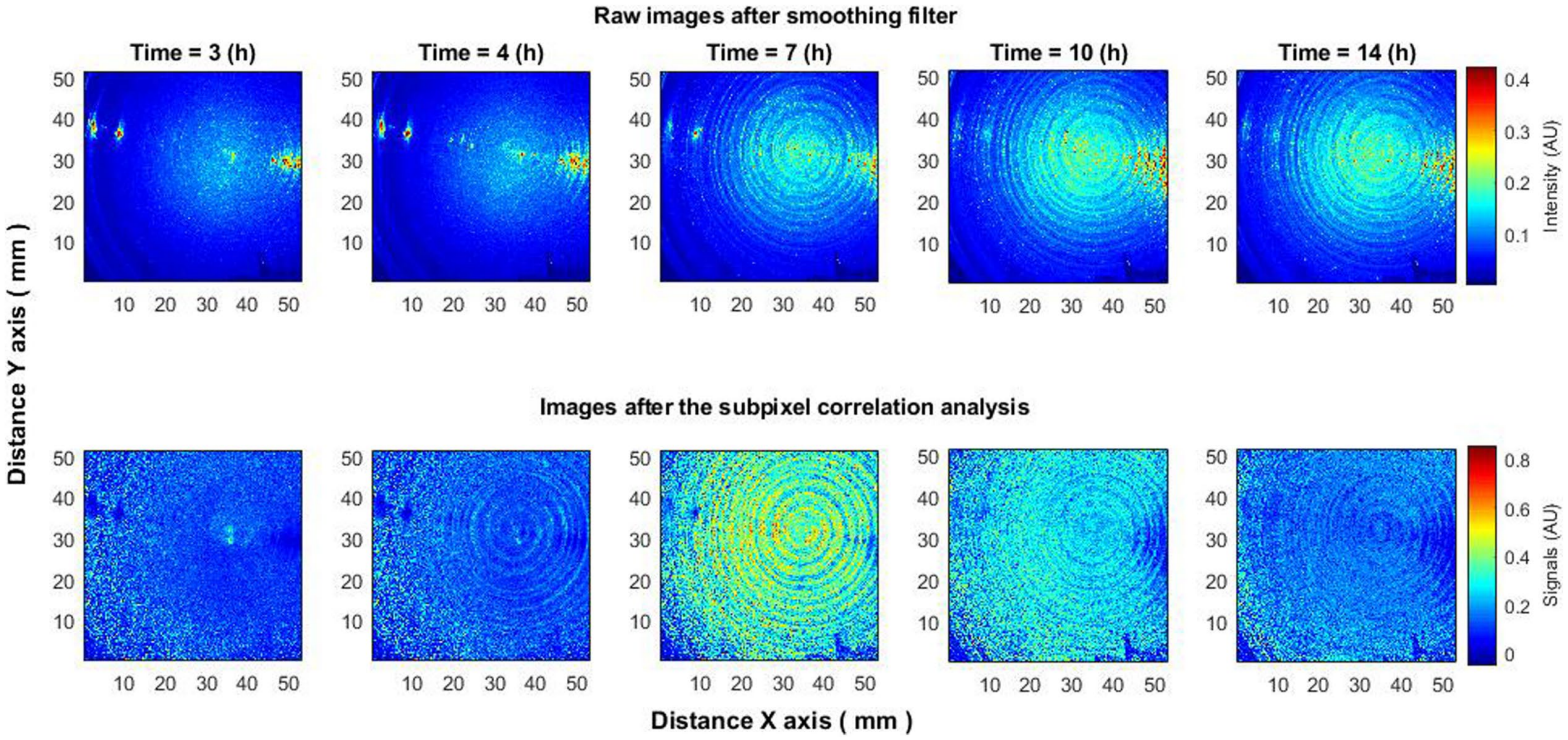
Bacteria *Klebsiella pneumoniae* put on the entire Petri dish. The subpixels correlation analysis of laser speckle images (bottom row) detects the appearance of bacteria earlier than raw speckle images (top row). Also increase (7 h) and decrease (14 h) of activity using subpixels correlation analysis can be observed, which is not visible on raw speckle images.

The observed behavior was consistent across different experiments and bacterial species such as *E. coli* and *S. aureus*. Additional experiments were conducted where bacteria were dispersed across an entire Petri dish, covering species like *Klebsiella pneumoniae*, *Enterobacter cloacae*, and *Staphylococcus aureus*. Using either raw or minimally processed speckle images (after spatial smoothing), the appearance and spread of bacteria could be observed. However, subpixel correlation analysis offered further insights: (1) it detected the presence of bacteria earlier than could be seen in raw speckle images (Figure 5, 4 h); (2) it captured a peak in activity that was not visible in raw images (Figure 5, 7 h); and (3) it observed a decrease in activity that also was not visible in the raw images (Figure 5, 14 h). This behavior was consistent across all tested bacterial species, suggesting that the observed effects are neither random nor isolated but constitute a recurring phenomenon applicable across different bacteria.

In a separate experiment, an antibiotic disk was placed on a Petri dish already exhibiting visible bacterial growth, as detected by the subpixel correlation method. This deviates from the standard method (The European Committee on Antimicrobial Susceptibility Testing, 2023), which introduces the antibiotic into a medium without observable bacterial growth. From previous work, it is known that bacterial activity is detectable 3–4 h post-inoculation using the subpixel correlation method (Balmages et al., 2021b). Therefore, the antibiotic disk was introduced into the Petri dish 4–4.5 h after bacterial inoculation (Balmages et al., 2023c). This approach allowed for real-time monitoring of the antibiotic’s effect on an already growing bacterial population.

**Figure 6:**
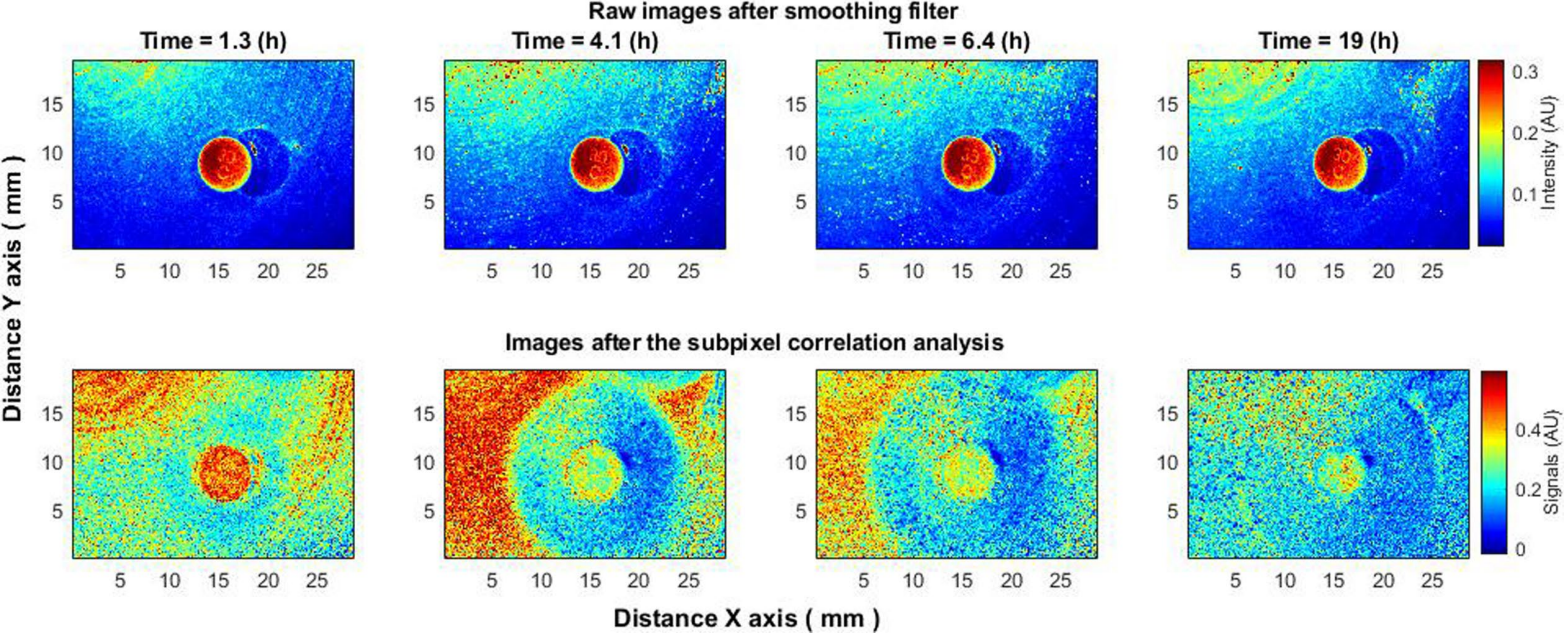
An experiment where the antibiotic disk was placed on the surface with bacteria already growing and clearly visible using the proposed subpixel correlation method. Subpixel correlation analysis of laser speckle images (bottom row) as opposed to raw speckle images (top row) immediately begins to demonstrate changes caused by the formation of a zone of inhibition.

The use of raw speckle images can indeed identify the presence of bacteria, but it falls short in providing immediate information about the formation of a zone of inhibition due to the antibiotic’s effect. The zone only becomes apparent hours later when new layers of bacteria appear outside this zone, but not within it. In contrast, the subpixel correlation analysis of laser speckle images starts showing changes indicative of a forming zone of inhibition almost immediately. This zone becomes distinct within 1.5 h or even sooner (Figure 6), providing a more timely and sensitive measurement.

Moreover, like previous experiments, the bacterial activity displayed a trend over time—peaking at around 4–5 h and almost ceasing by 19 h. More details on the analysis of the zone of inhibition using subpixel correlation methods can be found in the cited literature (Balmages et al., 2023c; Lihacova et al., 2023).

Therefore, the antibiotic experiment further corroborates our method for differentiating between active and inactive zones. This is accomplished by using two types of data: (1) laser speckle imaging without subpixel correlation, which merely identifies the existence of a colony without indicating its activity status; and (2) laser speckle imaging enhanced with sensitive subpixel correlation, which can specify whether the colony is growing or not.

### 3.3. Overall evaluation of the growth-related activity within the colony

**Figure 7:**
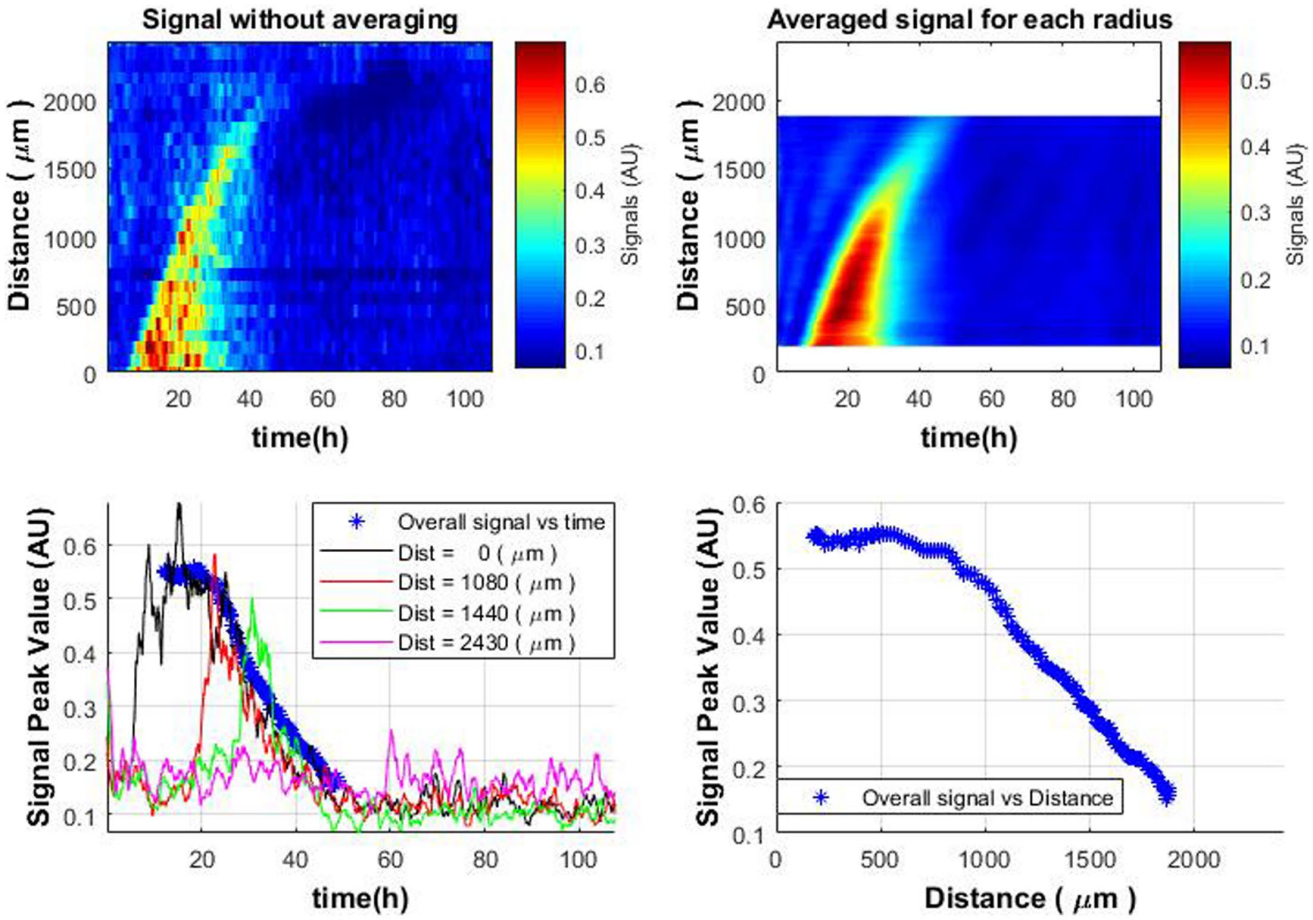
Spatiotemporal distribution of the average signal envelope for colony radius (top-right); spatiotemporal distribution without averaging (top-left); signal dynamics over time at various distances from the colony center compared to average signal envelope (blue bold stars curve, bottom-left); behavior of the average signal envelope vs. distance (bottom-right).


Observing that the colony is growing in the circle shape, in order to see the overall trend (and not only a cross-section along one of the axes, as shown in Figure 3) and reduce the influence of noise, the following analysis have been done:

1. The average of the signal envelope performed over each radius around the entire colony. It can be observed that the signal has become smoother (Figure 7 top-right vs. top-left, respectively).
2. The beginning of the decrease in activity throughout the colony occurs after the signals reach the peak values. Therefore, peak values were found for all signal envelopes in space (throughout the colony) as a function of time [Equation (6)],

(6) $$Env_{\left(\mathrm{peak}\;\mathrm{activity}\right)}\left[x,y\right]=\max_{n}\left(Env\left[x,y,n\right]\right)$$

where *n* is the time index.

The times when signal envelopes reach these peaks [Equation (7)],


(7) $$\mathrm{Time}\;\mathrm{In}d_{\left(\mathrm{peak}\;\mathrm{activity}\right)}\left[x,y\right]=\underset{n}{\mathrm{argmax}}\left(Env\left[x,y,n\right]\right)$$

and their location relative to the center of the colony [the radii; Equation (8)].


(8) $$\mathrm{Rad}\left[x,y\right]=\sqrt{{\left(x-x_{\mathrm{centr}}\right)}^{2}+{\left(y-y_{\mathrm{centr}}\right)}^{2}}$$

For each time [Equation (7)] and radius [Equation (8)], the median of the obtained values is performed [Equation (9)].


(9) $$\begin{array}{l} \hat{Env}\left[\mathrm{Time}\;Ind,\mathrm{Rad}\right]=\mathrm{median}\left(Env_{max}\left[x,y\right]\right)\;;\\ \;for\;n=\mathrm{Time}\;Ind,r=\mathrm{Rad} \end{array}$$

This operation is performed because there is a spread in these values, as colony growth depends on various factors (availability of oxygen and nutrients, Brownian motion, push and/or pull from neighboring cells in the colony, asynchrony of cell growth, proliferation in the colony, etc.). These results in two curves: (1) “overall” signal as a function of time and (2) “overall” signal as a function of distance from the colony center.

When analyzing the overall obtained spatiotemporal curve and the obtained signals separately in one graph, a coincidence can be observed (Figure 7 bottom-left). It is possible to detect changes in the signal level over time, which are similar to the pattern of bacterial growth within the colony. Thus, it is possible to evaluate the growth of the colony and identify whether the colony is active or not.

The observed decline in microbial activity as one moves away from the colony’s center (Figure 7 bottom—right) is an intriguing finding. It aligns well with the notion that microbial colony growth is self-limiting over time, likely due to the depletion of essential nutrients in the media. This is consistent with our earlier observations regarding microbial colony growth dynamics (Balmages et al., 2021b, 2023b), and it also aligns with existing mathematical models describing such growth behavior (Pirt, 1967; Rivas et al., 2014). Therefore, this additional insight into spatial variability in microbial activity within the colony further substantiates the utility of using laser speckle imaging in conjunction with subpixel correlation for a more nuanced understanding of microbial growth patterns.

### 3.4. Automatic classification between growing and non-growing bacterial colonies using artificial neural network

This section demonstrates the ability to classify between active and inactive bacterial colonies according to the features described in the previous sections.

The training set is diversified, featuring 10 different colonies spanning three bacterial species and experiments conducted on different days. The duration of growth in these training experiments varies from 25 to 68 h, providing a good range of data for machine learning. The decision to use *V. natriegens* colonies with a longer observation period of 107 h for the test set is also noteworthy.

Dividing the *x*-*y* field into small N × N (10 × 10) pixel sections and applying two different algorithms—one for pixel spatial averaging and the other for subpixel correlation—adds layers of complexity and data richness to analysis. By further segmenting these signals into time windows of 3 h, allows a finer temporal resolution for classification tasks.

The aggregate data size is large enough, encompassing thousands of signals for each colony in the training set. Given that we have 10 colonies in the training set, the overall sample size becomes statistically significant. This allows creating a robust and reliable machine-learning model capable of classifying active and inactive bacterial colonies based on the feature set described.

The decision to employ a Multilayer Perceptron (MLP) given the signal-based nature of the data seems pragmatic, especially considering the ease of implementation and computational requirements compared to other more complex models like CNNs or RNNs. The forward-looking approach to potentially comparing the effectiveness of different neural network architectures in future studies is a solid plan.

**Figure 8:**
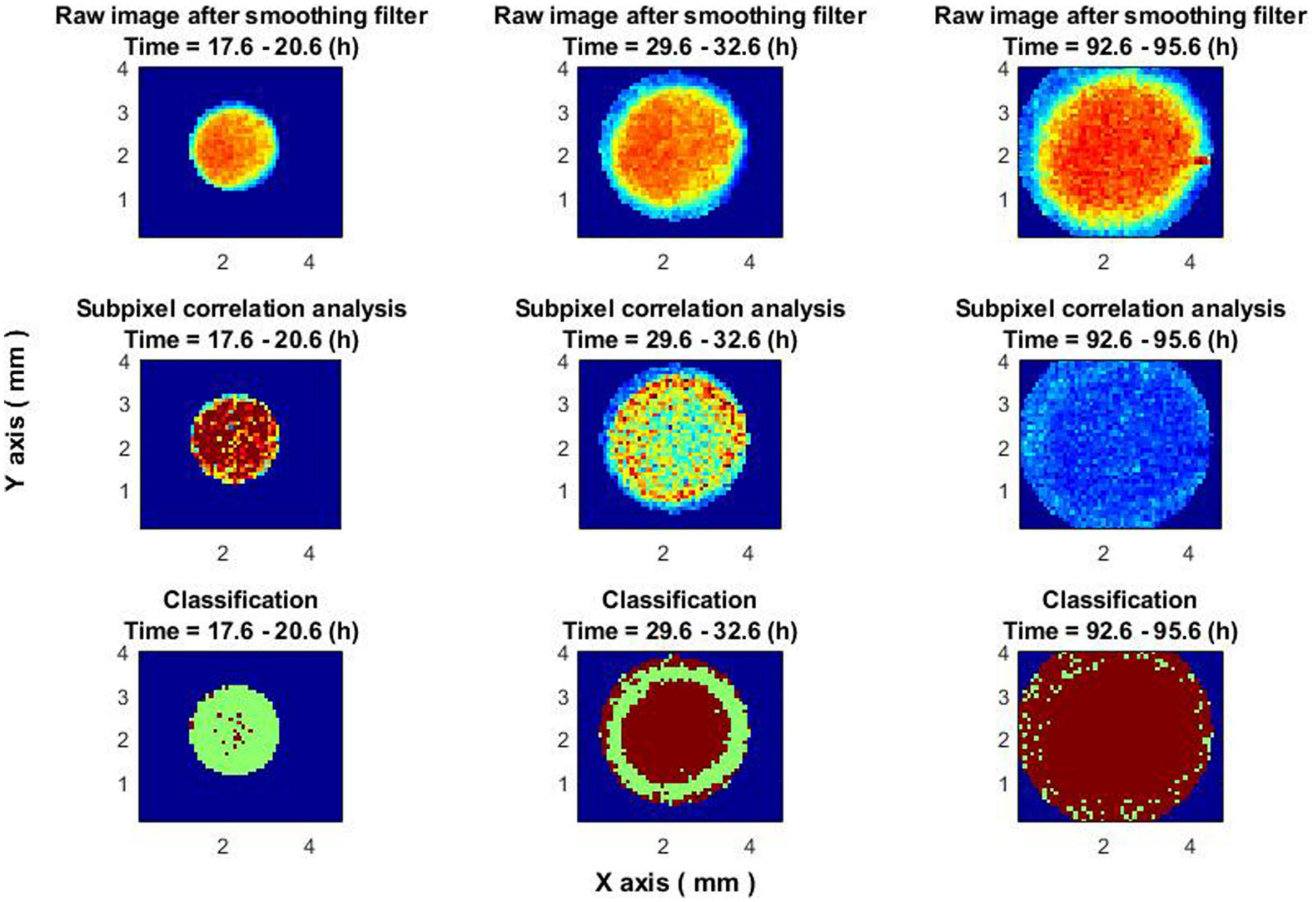
Classifier performance at different times. The images on the top row are raw images of colony after a smoothing filter. Central images—subpixel correlation analysis. Images on the bottom—classification result. In all images, the blue color is the out of colony region. In the bottom images (classification result), the result of non-growing zones is in brown, and the results of growing zones are in green.

Time-sliced classifier performance, illustrated in Figure 8, adds another layer of depth to the study. The ability to discern the evolving “ring” formation, its peak activity, and its eventual disappearance could offer substantial insights into bacterial colony growth dynamics. The time-resolved classification should be especially useful for understanding the stages at which colonies are most active and the transition points between activity and inactivity.

This methodology could have broader applications in both research and practical fields, such as monitoring bacterial resistance to antibiotics or assessing the efficacy of novel antimicrobial compounds.

## 4. Summary and discussion

The range of optical techniques available for studying bacterial colonies is indeed vast, each with its own set of advantages and limitations. While conventional approaches like direct microscopy and bright-field imaging (Bär et al., 2020; Sartori et al., 2021) provide valuable data on colony morphology and overall growth, they often fall short in capturing the dynamic activities within a growing bacterial colony. Techniques like Raman spectroscopy, although useful for metabolic analysis and bacterial identification, also have their limitations (Bae et al., 2016; Rodriguez et al., 2023). As an alternative approach to bacterial growth analysis, highly sensitive sensor-based methods employing microcantilevers for microbial detection are available (Etayash et al., 2016). However, these methods have constraints concerning spatial coverage and are suitable solely for the analysis of limited volumes of the bacterial incubation medium.

Among other methods able to register the growth of bacterial colonies, laser speckle imaging techniques stand out for their ability to capture micro-motions indicative of bacterial activity (growth). This sensitivity makes them uniquely capable of monitoring bacterial growth and behavior at a finer scale. Such precision is especially invaluable when studying microbial activity in the context of chemical screenings or antimicrobial susceptibility tests or impact of compounds on colony aging is essential (Váchová et al., 2012; Hashuel and Ben-Yehuda, 2019). Moreover, the technique’s potential for real-time tracking could revolutionize the way bacteriophage-bacterial interactions are studied, providing empirical data that could complement current experimental data and enhance mathematical models (Eriksen et al., 2018).

The advantages of laser speckle imaging, particularly when enhanced with subpixel correlation methods, make it a compelling tool for a variety of applications. These ranges from in-depth studies of bacterial growth dynamics to the exploration of microbial resistance mechanisms, potentially providing transformative insights into microbiological research.

The study sets out to showcase the utility of laser speckle imaging in discerning between active and inactive regions within bacterial colonies-something unattainable through traditional, marker-free growth observation methods using white light illumination. By applying subpixel correlation analysis, the method is finely tuned to detect subtle changes in the object under observation, effectively distinguishing colonies where bacteria are still proliferating colonies from dormant or dead colonies.

The proposed laser speckle imaging technique has the potential to bring novel research technique in biotechnological processes. By enabling *in situ* observations, the method allows for real-time monitoring of bacterial responses to various chemicals and potential inhibitors. This opens the door for more dynamic, responsive experimentation, creating new opportunities for advancing the field.

Looking ahead, incorporating laser speckle imaging into existing high-throughput platforms and advancing data processing algorithms hold the promise of broadening the application spectrum of this innovative technique. The method could become instrumental in various research domains, from studying bacterial growth dynamics to evaluating the efficacy of antimicrobial substances.

## 5. Conclusion

The addition of subpixel correlation analysis to laser speckle imaging offers a nuanced understanding of bacterial colony activity that is not achievable with either laser speckle or white-light imaging alone. This analytical layer enables the detection of subtle changes in colony activity and offers a mechanism for distinguishing between growing and non-growing bacterial colonies. Such capabilities pave the way for deeper studies into the complex interactions occurring within microbial communities. Ongoing research aims to extend this classification to zones with differing bacterial characteristics, enhancing the method’s utility in both fundamental and applied microbiology. In fields where understanding the intricacies of colony behavior is critical—such as physiology, genetics, or the study of environmental impacts on growth—this refined imaging technique promises to offer invaluable insights.

## Data availability statement

The raw data supporting the conclusions of this article will be made available by the authors, without undue reservation.

## Author contributions

IB: Conceptualization, Data curation, Formal analysis, Investigation, Methodology, Resources, Software, Visualization, Writing – original draft, Writing – review & editing. JL: Conceptualization, Data curation, Formal analysis, Funding acquisition, Investigation, Methodology, Resources, Supervision, Writing – original draft, Writing – review & editing. SZ: Conceptualization, Data curation, Formal analysis, Investigation, Methodology, Writing – review & editing. DB: Conceptualization, Data curation, Formal analysis, Funding acquisition, Investigation, Methodology, Project administration, Resources, Software, Visualization, Writing – review & editing. RB: Conceptualization, Data curation, Formal analysis, Investigation, Methodology, Writing – review & editing. IL: Conceptualization, Data curation, Formal analysis, Investigation, Methodology, Supervision, Writing – original draft, Writing – review & editing. AL: Conceptualization, Data curation, Formal analysis, Funding acquisition, Investigation, Methodology, Project administration, Resources, Supervision, Writing – original draft, Writing – review & editing.
